# Fractures of the Maxillæ

**Published:** 1890-11

**Authors:** Will. H. Whitslar

**Affiliations:** Youngstown, O.


### COMMUNICATIONS.

### Fractures of the Maxillæ.

WILL. H. WHITSLAR, D.D.S., M.D., YOUNGSTOWN, O.

Read before the Mahoning County Medical Society.

Of all arthrodial joints and their muscular attachments there is no joint so important to its possessor as the tempero-maxillary articulation. At this joint in common with its corresponding joint swings the pivot of mastication and conversation. The Creator has placed as a necessary factor what man has denominated as "muscles" to control the movements of the denser parts of the body which go to make up the articulation.

It is of these denser portions that we are to pay direct attention this evening, and yet, without a perfect understanding of the soft tissues surrounding the bones nothing like a correct approximation of the normal condition can be made when, by some cause, the parts are found broken in continuity.

The best results in the treatment of fractures of the maxillæ necessitates a comprehensive study of the normal relations of the teeth and jaws. It is important to know that while one set of muscles may depress a broken fragment of jaw, another may elevate it. It will not be amiss to recall to mind the muscles that may or may not be troublesome agents in the reduction of fractures of the maxillary bones.

The muscles attached to the superior maxilla have rarely any thing to do separately with fractures, but all the muscles grouped together render the usual aid in drawing the fragments back *in situ*. The superior maxillary bone is so vascular that repair is soon accomplished. It is not necessary therefore to consider these muscles separately, but make the general statement that when a fracture of the upper jaw is found, it is usually one produced by an object falling so as to depress the lateral halves of the maxilla in a direction from the resisting borders of the bones in contiguity, and that pressed into the original position, the parts are held without difficulty until they are united.

The tempero-maxillary articulation permits a variety of movements owing largely to the form of the articulating surfaces, and also the comparative absence of retaining ligaments, and hence we find movements unusual in any other than a ball and socket joint. In closing the jaw the rotary and oblique motions are accomplished by four pairs of very powerful muscles, and these are antagonized by muscles feeble in strength, and indirect in their application. The masseters and temporals are attached to the outer sides of the jaw, and the external and internal pterygoids to the inner sides constituting the four pairs of muscles previously spoken of.

"The temporal, masseter, and internal pterygoid raise the lower jaw against the upper with considerable force." It is said that in ordinary biting an average force of fifty pounds is exerted. Thus you may perceive that whilst in a state of active contraction the force is very great, and it may be argued that the muscles being large to produce such force, their natural muscular contraction or tension when inactive must also be great in proportion to any one of the smaller muscles, and hence, artificial forces guided by man's supervision, aids the small muscles, opposing the large ones in their action in correcting irregularities.

To further study the mechanism of the jaw and its movements Gray's Anatomy describes the action of the muscles as follows: "The superficial portion of the masseter and the internal pterygoid assist the external pterygoid in drawing the lower jaw forward upon the upper, the jaw being drawn back again by the deep fibres of the masseter and posterior fibres of the temporal. The external pterygoid muscles are the direct agents in the trituration of the food, drawing the lower jaw directly forward, so as to make the lower teeth project beyond the upper." This gives the appearance which we call the "under-hung" jaw. "If the muscles of one side acts the corresponding side of the jaw is drawn forward and the other condyle remaining fixed, the symphysis deviates to the opposite side. The alternation of these movements on the two sides produce trituration."

The other muscles which may have to do with fractures of the maxillæ are the digastric, mylo-hyoid, genio-hyo-glossus; the platysma myoides is also, sometimes an important muscle to control, but mention of it in treatises on fractures of the jaw is very limited.

From the foregoing it can be easily seen that control over muscular contraction is a great hinderance toward getting and keeping fractured bones in proper position.

The human jaw-bone is the most difficult bone to hold in apposition during the process of healing.

From what causes may fractures of the maxillæ result? They are usually the result of direct violence. Prof. Pancoast described a case in which fracture of the neck of the bone resulted from a violent fit of coughing. "Fractures of the lower jaw are remarkable from the fact that they are almost always *compound* toward the mouth, though the skin is rarely involved except in gunshot injuries. The fibrous tissue of the gum being very inelastic tears readily when the bone is broken across, and thus the saliva and the air come in contact with the fractured surfaces. This statement only applies, however, to fractures of the body of the bone, for when the ramus, or still more, when the coronoid process or condyle is broken, the bone is too deeply seated for the injury to extend into the mouth."

Fracture of the lower jaw may occur at various points, but the body of the bone is the part usually broken. Except at the symphysis the line of fracture is usually oblique.

Symptoms.—It would be almost a waste of time to describe to you the ordinary signs of a broken bone, but yet I have seen physicians unable to directly diagnose a fractured maxilla. It may, however, baffle the best surgeon to understand some fractures of the condyle, its neck, and the ramus, while those of the body of the jaw are easy to detect.

Complications.—The complications attending fractured maxillæ may be wounds of the mouth or face, hemorrhage, paralysis, neuralgia, abscesses, salivary fistulæ, convulsions, necrosis, and dislocation of the condyle from the glenoid cavity. From these complications serious results may intervene unless the attendant is fully prepared to cope with the case. Hence, the necessity of a thorough knowledge of all the parts and their workings.

Fractures of the jaws require mechanical contrivances, or supports, to retain the fragments in position. Fractures of the jaws are not a modern inconvenience, but evidences of such may be found in Biblical history. Perhaps the first surgical aid in support of fractured bones was with bandages. Hippocrates mentions the use of bandages. Though fractured maxillæ usually fall into the hands of the general surgeon, yet the best appliances that have been invented have emanated from dentists. These appliances now are many, and they all serve a good purpose though no one piece of mechanism will meet every case.

In complicated cases government is dependent upon the peculiarities that are presented. Interdental splints are the means that are now most frequently used in the treatment of fractured jaws. The interdentel splint was first used by Chopart and Desault in 1780. Splints may, in cases of emergency, be made of gutta-percha, and sometimes these may be made use of permanently. Vulcanized rubber makes an admirable splint when used after the form used by Dr. Norman Kingsley, of New York. The wire splint devised by Mr. Hammond, L.D.S., of England, is also a useful splint. A specimen of each of these splints I have the pleasure of showing you this evening, together with models and the new system of regulating teeth and holding fractured jaws in place, by the plan originated by Dr. Edward Angle, of Minneapolis, Minn. This system of Dr. Angle's is one of the most complete and satisfactory methods of holding the broken jaw in place where there are teeth to fasten the splint to that has yet been devised, and I invite your careful study of the models that are represented for illustration and your study.

